# Effect of linear accelerator carbon fiber couch on radiotherapy dose

**DOI:** 10.1371/journal.pone.0277332

**Published:** 2022-11-08

**Authors:** Fantu Kong, Meiting Lu, Jie Dong, Donghui Wang, Junyue Shi, Zhenghuan Li

**Affiliations:** 1 The Third Affiliated Hospital of Sun Yat-sen University, Guangzhou, China; 2 Foresea Life Insurance Guangzhou General Hospital, Guangzhou, China; 3 China Institute of Atomic Energy, Beijing, China; Pisa University Hospital, ITALY

## Abstract

This study aimed to explore the effect of carbon fiber couch on radiotherapy dose attenuation and gamma pass rate in intensity-modulated radiotherapy (IMRT) plans. A phantom inserted with an ionization chamber was placed at different positions of the couch, and the dose was measured by the chamber. Under the same positioning, the phantom dose was calculated using the real and virtual couch images, and the difference in the planned dose of radiotherapy was compared. Ten clinical IMRT plans were selected as dose verification data, and the gamma pass rates were compared between couch addition and non-addition conditions. When the radiation field was near 110° and 250°, the measured value attenuation coefficient of the ionization chamber at the joint of the couch was up to 34%; the attenuation coefficient of the treatment couch from the actual couch image calculated using the treatment planning system (TPS) was up to 33%; the attenuation coefficient of the virtual couch calculated using the TPS was up to 4.0%. The gamma pass rate of the dose verification near gantry angles 110° and 250° was low, and that of the joint could be lower than 85% under the condition of 3%/3 mm. The gamma pass rates of the radiation field passing through the couch were all affected. The dose was affected by the radiation field passing through the couch, with the largest effect when passing through the joint part of the treatment couch, followed by that of the main couch plate and extension plate. When the irradiation field passed through the joint and near 110° and 250° of the main couch, the dose difference was large, making it unsuitable for treatment.

## Introduction

With the widespread application of modern intensity-modulated radiotherapy (IMRT), the industry’s requirements for the accuracy of radiotherapy dose are also increasing. ICRU report No. 24 showed that to ensure the quality of a radiotherapy plan, the overall dose deviation from the primary tumor needed to be controlled within 5% of the tumor territory; otherwise, recurrence or complications may occur [1]. Simultaneously, the AAPM TG 53 report concluded that the dose calculation error of the radiotherapy treatment planning system (TPS) should not exceed 3% [2]. To ensure the accuracy of the dose calculation of the TPS, systematic and standardized sampling and calibration of the linear accelerator must be performed before use, and the TPS output model must be fitted using the real dose output of the linear accelerator. Although the accuracy of the TPS can be guaranteed by the linear accelerator manufacturing corporation’s calculation model fitting, there are many factors involved in the design of the radiotherapy plan that affects the dose delivery implemented, such as the treatment couch system that supports the patient. The widely used linear accelerator treatment couch system is rigidly connected to the treatment couch, made of carbon fiber, through a movable base, which has the advantages of high hardness, lightweight, and low radiation attenuation. However, the TPS does not consider the effect of the actual treatment couch on the planned dose calculation. Specifically, computed tomography (CT) image positioning of the treatment couch is not established; therefore, the plan designer cannot add the actual treatment couch to the plan image to be calculated on the TPS. Coupled with treatment fractions at different positions, this uncertain factor continues to affect the accuracy of the actual treatment plan dose delivery.

The physical structure of the treatment couch can be divided into three parts: the main couch, an extension plate, and a joint mechanism between them. Although the carbon fiber material causes low attenuation of the rays, a dose error remains [3–6]. Due to the large attenuation of the radiation at specific angles and positions of the treatment couch [7], without suitable couch modeling, the dose received by the patient during the treatment process is lower than the planned dose, reducing treatment effectiveness. Studies have shown that the treatment couch can increase the dose to the skin and may cause serious damage [8–10]. With the widespread use of IMRT technology, the complex multileaf collimator (MLC) motion puts forward higher requirements for the verification of the planned dose. The IMRT plan must undergo dosimetry verification before clinical treatment to ensure the accuracy of the treatment plan. However, only limited reports on the verification of plans have considered the presence of the treatment couch. Some of them have incorporated a virtual treatment couch model in the treatment plan design, showing that the deviation of the planned dose can be reduced [11]. However, positioning errors in fractions exist and the treatment couch structures vary; therefore, the virtual treatment couch cannot fully simulate accurate dose attenuation. A combination of a carbon fiber treatment couch and a support base plate for the body position fixation device may affect dose verification results, leading to a significant decrease in the gamma pass rate in the plan for nasopharyngeal cancer (NPC) [12]. Consequently, targeted dosimetry studies are required on the treatment area falling on different positions of the couch and different angles of ray incidence.

Therefore, this study aimed to investigate the effects of high-energy X-rays with different angles of incidence and different positions of the treatment couch on the planned dose of radiotherapy. A phantom inserted with the air ionization chamber was placed in four different positions on the treatment couch, and the couch was moved so that the ionization chamber was at the isocenter. The dose values in the designated area at the center of the phantom at several angles of incidence were measured and calculated, and statistical and comparative analyses of dose differences were performed using an electronic portal imaging device (EPID) to confirm pass rates. This evidence may support accurate calculation and dose correction of the radiation dose in the later stage and may help reduce the effect of the treatment couch on the radiation dose.

## Materials and methods

### Equipment and materials

This research was performed using the Elekta Synergy linear accelerator, which was equipped with an iBEAM evo Couch carbon fiber treatment couch and an iBEAM evo Extension 415 extension plate, with dimensions of 200×53×5 cm^3^ and 41.5×53×2 cm^3^. Using a sandwich structure [13], the inner and outer layer materials were foam and carbon fiber, respectively. The thickness of the carbon fiber external layer was 1.2 mm. We used the planning system Elekta TPS (Monaco V5.11.03) to calculate the planned dose. Planning images were taken using the Siemens large-aperture positioning CT (SOMATOM Definition AS) and cone beam computed tomography (CBCT) scans from Synergy linear accelerator (LINAC). The dose analysis phantom used a QUASAR cylinder model with an internally nested finger ionization chamber, with a length of 18 cm, an outer diameter of 7.8 cm, and a detector opening at the center of the phantom. The dose measurement model was an SNC600c finger ionization chamber. Ten NPC IMRT plans (December 2020 to March 2021) were retrospectively selected for plan verification, and Raydose’s three-dimensional dose verification system Edose, which is an EPID-based system, was used as the equipment. All validation plan data contained only MLC control points, and physical dose information was obtained from the planning system and did not involve any patient data. The experimental results were statistically analyzed using SPSS 25.0 statistical software. First, an analysis of variance was performed. Data that met the homogeneity of variance assumption were subjected to the t-test, while the data that did not meet this assumption were subjected to the rank-sum test. P-values of <0.01 were indicative of statistically significant differences.

### Measurement of the attenuation of radiation by the treatment couch with an ionization chamber

**Fig 1:**
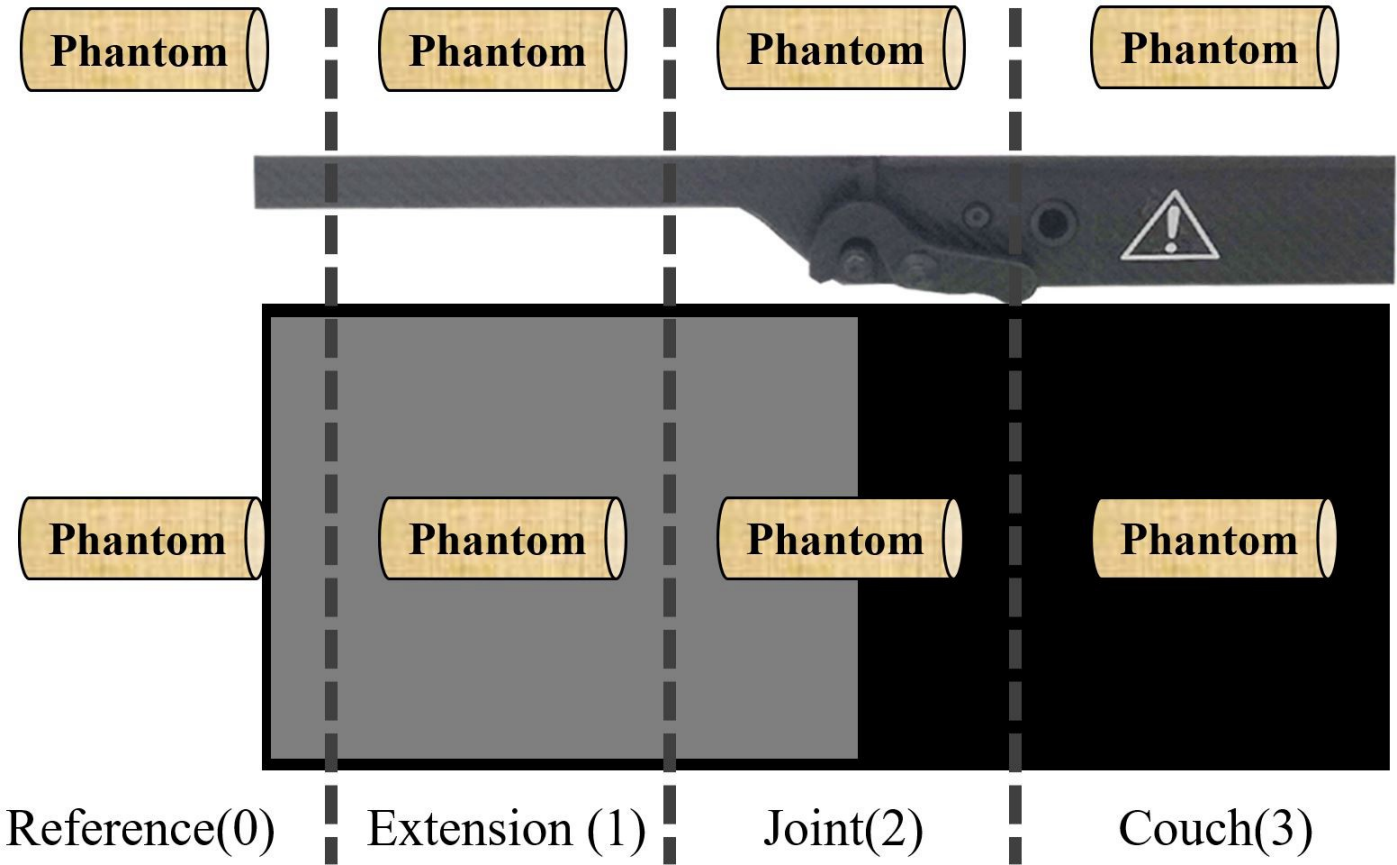
Location of the treatment couch with respect to the phantom that is placed on the treatment couch with the ionization chamber point of measurement at the isocenter.

The phantom was placed such that the effective point of measurement was at the isocenter. The lateral deviation of the treatment couch was set to 0 mm, and the couch was placed in four positions (Fig 1) such that the phantom was in the reference position (0), i.e., hanging in front of the treatment table, or placed over the extension plate (1), joint part (2), or main couch (3). There was no special fixing bracket for the phantom. We achieved a state where most of the phantom was suspended in front of the treatment couch by fixing the tail of the phantom so that the detector was not affected by the treatment couch. The angle between the accelerator collimator and treatment couch was 0°, and the field area was 10×10 cm; 6-MV X-ray beams were emitted, the gantry regularly emitted beams at intervals of 10° to emit 100 MU beams, and the dose rate was 600 MU/min. For each measurement condition, three measurements were taken and the obtained values were averaged. D_M0_ represented the measured value when the ionization chamber was at the reference position, while D_M_ represented the measured value of the ionization chamber at the extension plate, joint part, and main area of the treatment couch (D_M1_, D_M2_, and D_M3_, respectively). The ray attenuation coefficient δ_M_ (%) was calculated using the formula δ_M_ = (D_M0_-D_M_)/D_M0_×100% [14].

### Calculation of the radiation attenuation by the actual couch structure with TPS

**Fig 2:**
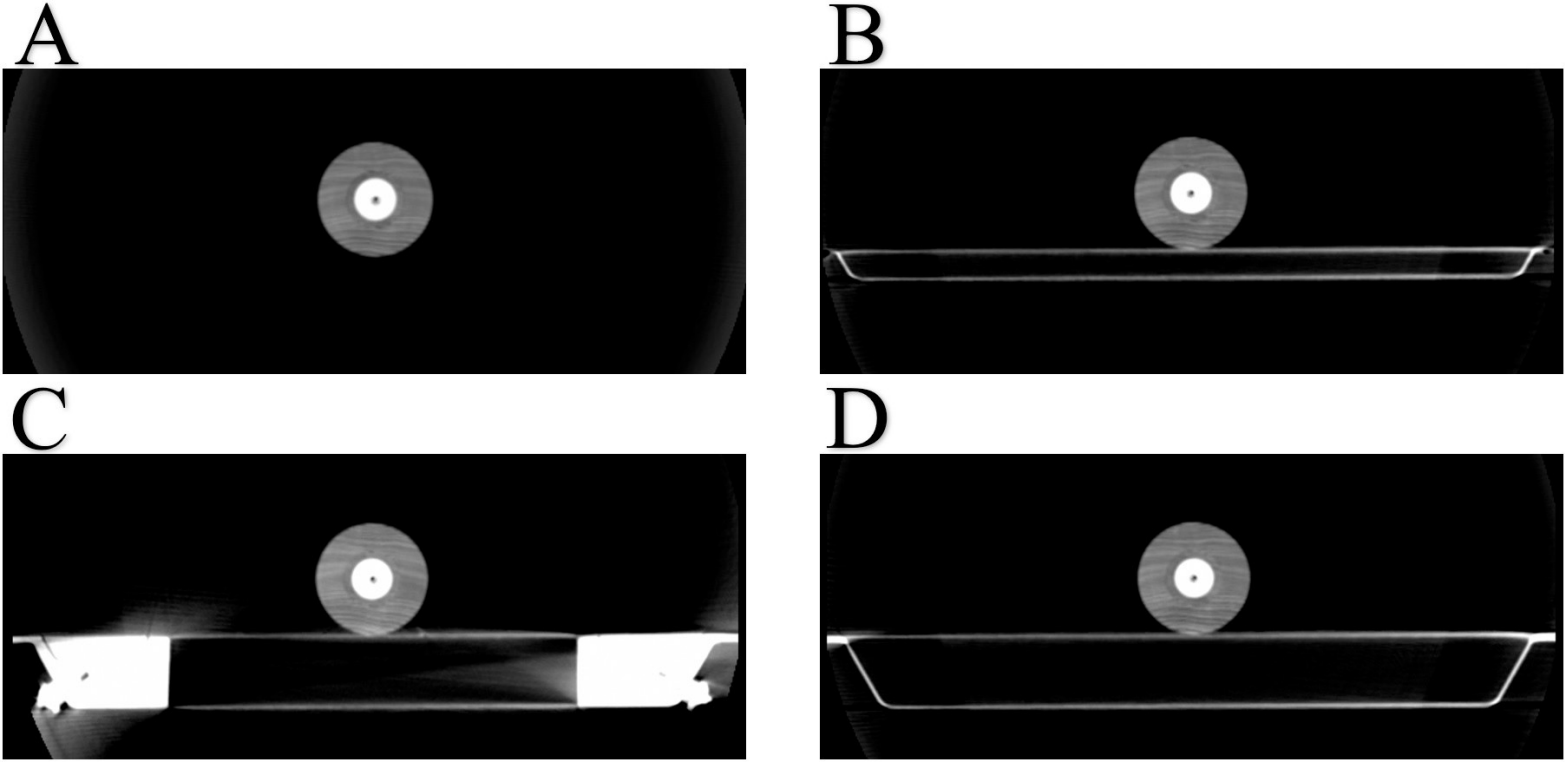
Panels A, B, C, and D present CBCT images of the phantom placed at the reference position (hanging in front of the treatment couch), extension plate, joint part, and main couch, respectively.

The position of the phantom was consistent with the measurement conditions presented. The scanned images of the CBCT system parameters (F1, L20) were adjusted and transmitted to the TPS, and the sensitive air of the ionization chamber was delineated as the region of interest. The aim of transmitting the images of the treatment couch at different positions in the CBCT scan to the TPS was to obtain the actual treatment couch structure at different positions and calculate its effect on the dose attenuation in the TPS. The LINAC treatment couch does not coincide with the CT scan couch; thus, CBCT is needed to determine the treatment couch image. In the TPS, a 10×10 cm irradiation field was set, and beams were emitted with 6 MV X-rays using the Collapsed Cone algorithm, a calculation grid of 0.1 cm, and a gantry interval of 10°. Fig 2A–2D present the CBCT images with the phantom placed at the reference site (hanging in front of the treatment couch), extension plate, joint part, and main couch, respectively; the calculation results of the corresponding positions are represented as D_CBCT0_, D_CBCT1_, D_CBCT,_ and D_CBCT3,_ respectively, where D_CBCT0_ represents the calculated value of TPS without the effect of the treatment couch. The formula δ_CBCT_ = (D_CBCT0_-D_CBCT_)/D_CBCT_×100% was used to compare the relative deviation between the actual measured value and the actual couch value calculated using the TPS.

### Calculation of the attenuation of rays by the virtual treatment couch model with TPS

Siemens large-aperture CT was used to scan the phantom with the ionization chamber inserted using a 500-mm field of view (FOV) scan condition, with a voxel size of 0.97×0.97×1 mm^3^. The image was then transmitted to the TPS. The TPS set up a 10×10 cm irradiation field, 6 MV X-rays, a Collapsed Cone algorithm, a calculation grid of 1 mm, and the gantry emitted beams of 100 MU every other 10°. The dose calculation was divided into two groups; the first group outlined only the contour of the phantom, while the second group added a TPS virtual couch model based on the first group. The sensitive air of the ionization chamber was outlined as the region of interest. Monaco considers for the attenuation only the voxels inside the external structure; thus, in the first group, in which only the phantom was contoured and its property was assigned as an external contour, the couch was not accounted for in any way. The formula δ_CT_ = (D_CT0_-D_CT1_)/D_CT0_×100% was used to compare the relative deviation of the two groups, where D_CT0_ represents the calculated value without adding a virtual couch and D_CT1_ represents the calculated value with the virtual couch. We used the virtual therapy couch model developed by the Monaco TPS and used the used the default relative electron density value of Elekta TPS, where the carbon fiber is 0.5 and the foam is 0.03.

### Plan verification results

**Fig 3:**
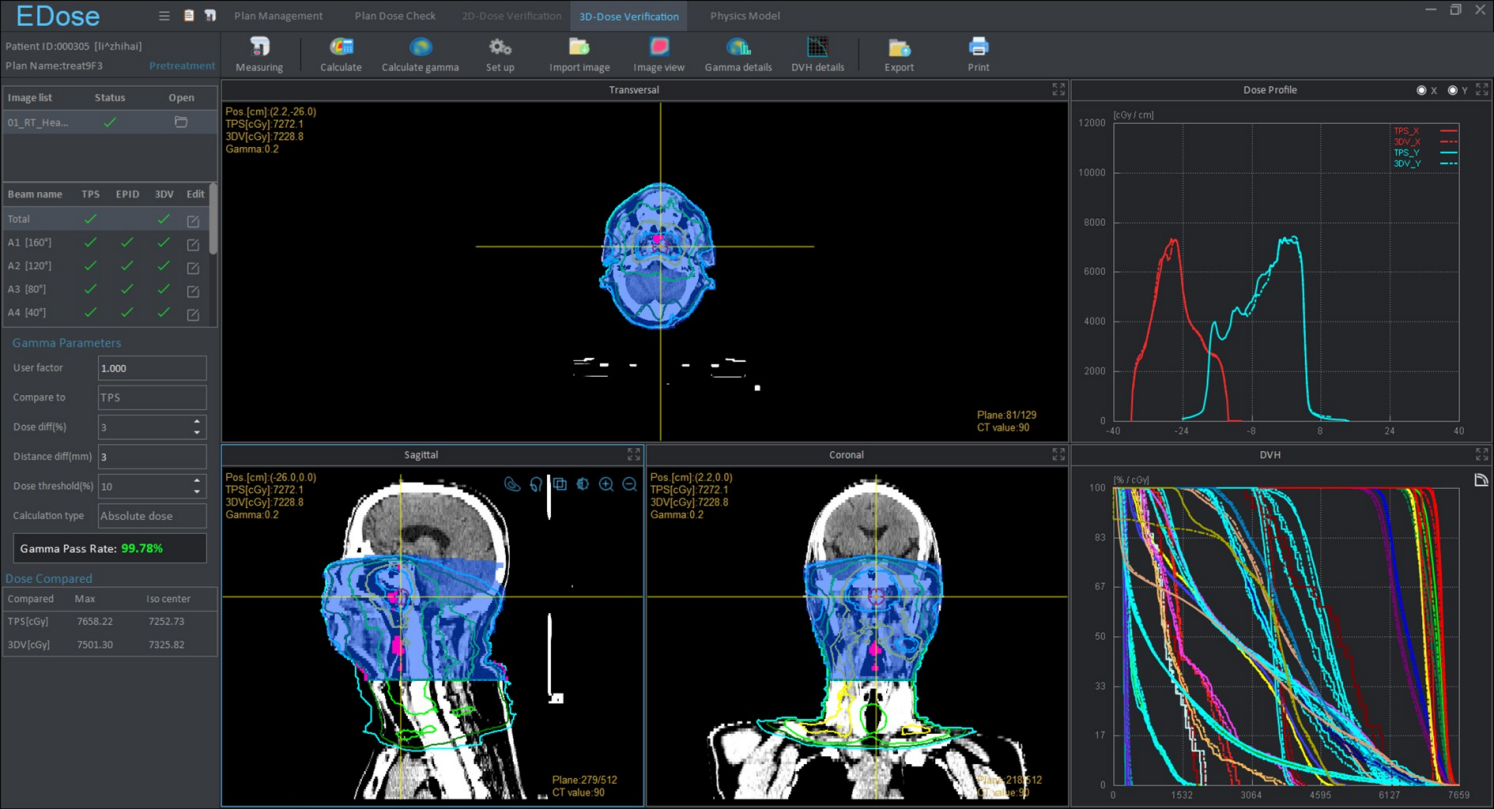
Pretreatment gamma pass rate calculation diagram constructed using Edose software.

Using the Edose dose verification calculation software (Fig 3) and a linear accelerator EPID system, the pretreatment gamma pass rate of 10 cases of NPC IMRT plans was verified to determine the effect of attenuation when X-rays passed through the treatment couch on the plan verification. The beams were emitted under four different positions of the planned isocenter point at the reference, extension plate, joint part, and center of the main couch, respectively, and the gamma pass rate before the planned treatment was measured. The 10 cases of clinical plans were nine-field intensity modulation plans. The gantry angles were 160°, 120°, 80°, 40°, 0°, 320°, 280°, 240°, and 200°, and the collimator angles were 0°. When the gantry angles were 80°, 40°, 0°, 320°, and 280°, the radiation field did not pass through the treatment couch before reaching the target area. In this study, the four gantry angles, namely 160°, 120°, 240°, and 200°, were compared. The gamma pass rate was calculated under the following conditions: 3% dose difference, 3 mm distance difference, 10% dose threshold, and calculation type as absolute dose.

## Results

### Ionization chamber measurement and TPS calculation results

**Fig 4:**
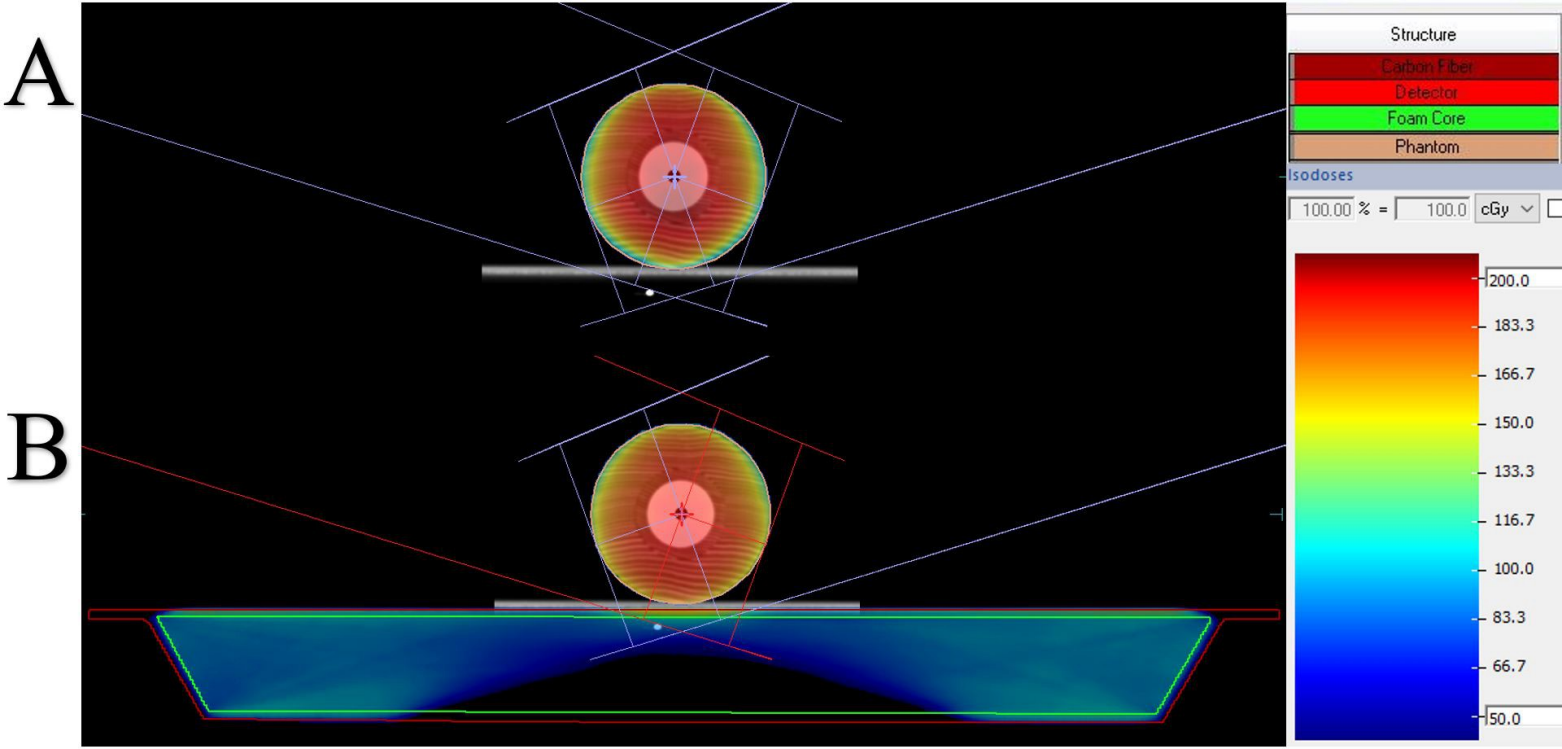
Panels A and B show the calculation diagram of the TPS dose before and after adding the treatment couch.

The results of the ionization chamber measurements, TPS of actual couch image calculations, and TPS of virtual couch effect calculations for each part of the directly added treatment couch are shown in Table 1. The dose measurement result is directly read by the ionization chamber, and the dose calculation result is the average value of the sensitive volume delineated area of the detector. The results of the phantom at the reference position were recorded as D_M0_, D_CBCT0_, and D_CT0_. The results were statistics for each dose in 36 gantry angles. The reference dose values in all groups were higher than the mean dose values after treatment couch attenuation (P≤0.01). The results show that the measured and calculated values have the largest attenuation at the joint, reaching values of 6.74% and 4.05% on average, respectively, followed by the main couch and extension plate, with an average attenuation of 2.5% and 1.5%, respectively. Fig 4A and 4B present dose distribution diagrams before and after adding the virtual treatment couch.

**Table 1:** Measurements calculated with the presence of a real couch, TPS calculation of actual couch based on CBCT images, and TPS calculation introducing a virtual couch model and corresponding P-values per group.

Group	M				CBCT				CT	
	0	1	2	3	0	1	2	3	0	1
Mean±SD	103.57±0.15 cGy	102.13±1.88 cGy	96.59±9.33 cGy	101.23±2.84 cGy	98.4±0.00 cGy	97.18±1.80 cGy	94.41±7.80 cGy	96.1±3.00 cGy	97.1±0.00 cGy	95.82±1.57 cGy
P	NA	<0.01	<0.01	≤0.01	NA	<0.01	<0.01	<0.01	NA	<0.01
δ (%)	NA	1.4	6.74	2.26	NA	1.24	4.05	2.34	NA	1.32

P-values and δ (%)-values are derived from comparisons between the measured and reference values of each group, such as M1 corresponding to the P-value representing P (M1 and M0).

### Relative deviation results

**Fig 5:**
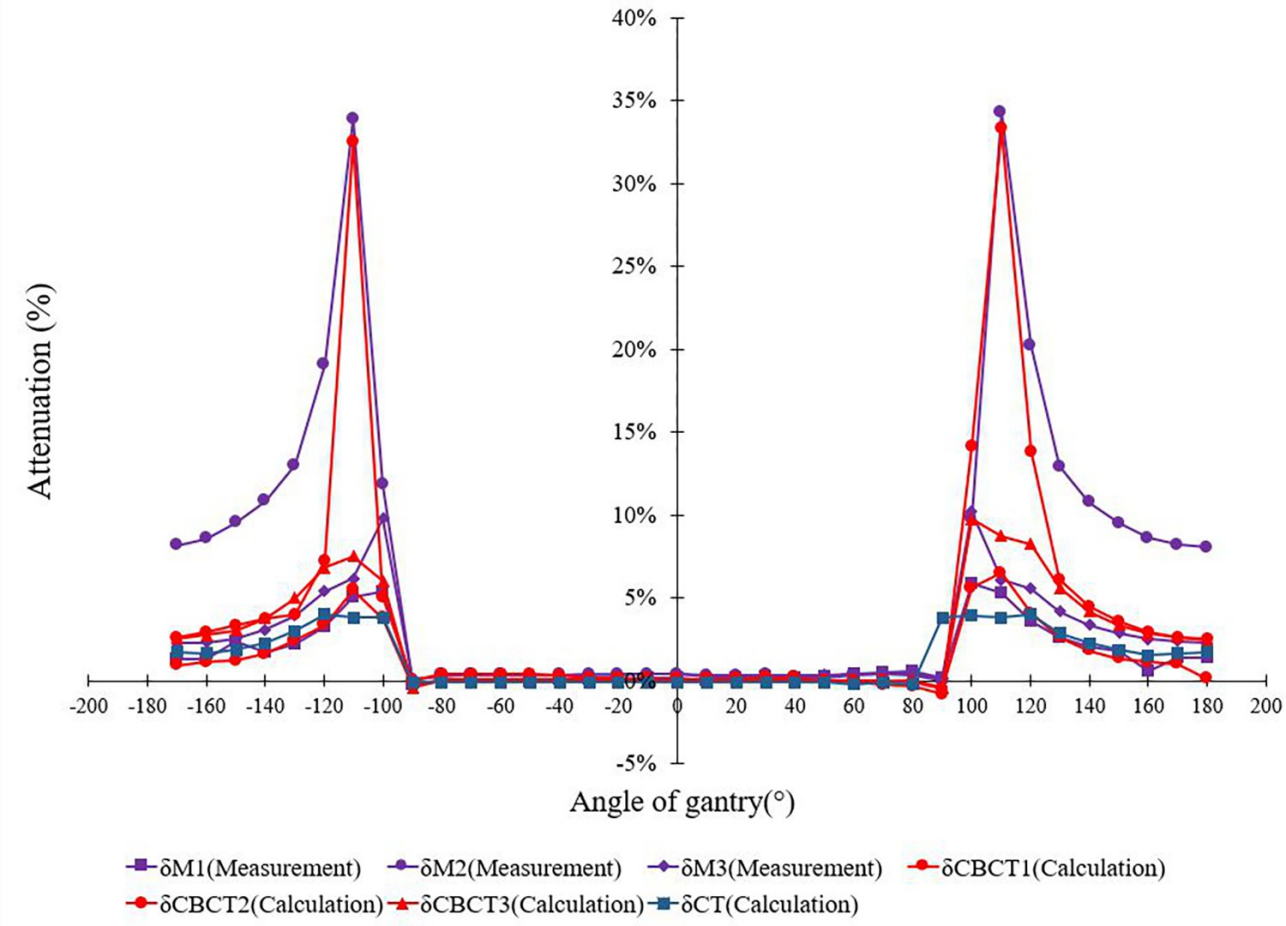
Attenuation factors δ changing with gantry angles under different conditions (M1: Extension; M2: Joint; M3: Couch; CBCT1: Extension; CBCT2: Joint; CBCT3: Couch; CT: Couch).

**Fig 6:**
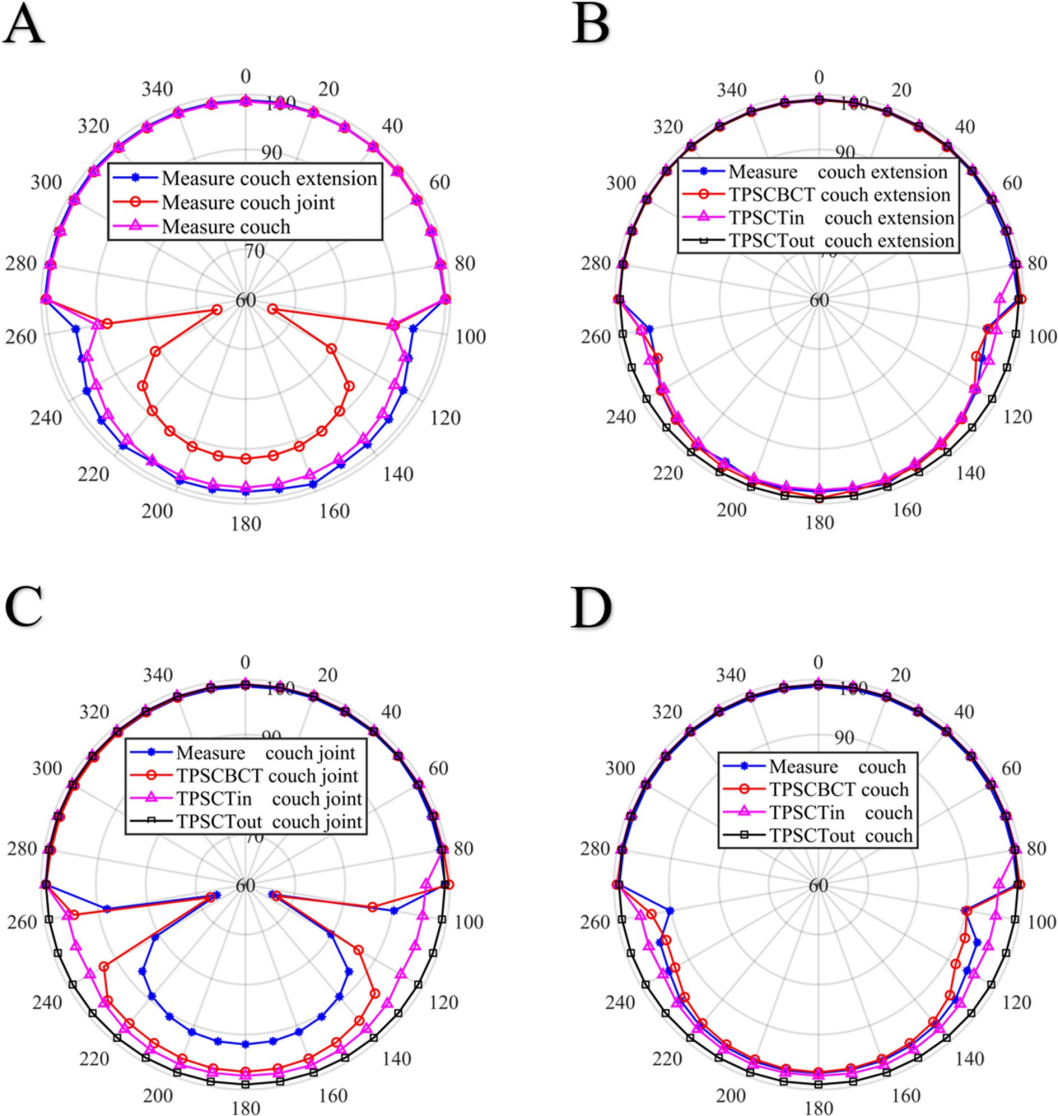
A: Measured dose values for the different attenuation cases. B, C, and D: Measured (M) and calculated values (CBCT, CT) for the extension, joint, and couch positions, respectively.

The results were statistics for each dose in 36 gantry angles. Fig 5 shows the measured results of the phantom that was placed such that the effective point of measurement was at the isocenter, the lateral deviation of the treatment couch was set to 0 mm, and the couch was placed in four positions (Fig 1). Therefore, the phantom was in the reference position, i.e., hanging in front of the treatment table, or placed over the extension plate, joint part, or main couch. Fig 6A shows the measured values of the detector at different positions, while Fig 6B–6D show the measured and calculated values of the detector at different positions. The measured values of the extension plate, joint, and main couch and TPS calculated of the actual couch dose were almost symmetrically distributed, and the dose distribution curves had obvious depressions at 110° and 250°, with the dose attenuation at the junction being much greater than at other positions.

The results showed that the error of the measured value and actual couch value, calculated by TPS, increased as the thickness of the irradiation field passing through the treatment couch increased. The thickness of the main couch where the irradiation field passed through near 110° and 250° was the largest; therefore, the measured value at the joint and the δ value of the actual couch calculated by TPS were the largest, reaching 34% and 33%, respectively. The virtual couch in the TPS had the smallest attenuation of the uniform model, which was 4%.

### Comparison of the pass rate of the plan verification

Table 2 shows the verification results of pretreatment gamma pass rates of 10 cases of patients with NPC undergoing an IMRT plan at 160°, 120°, 240°, and 200° irradiation field at the reference, extension plate, joint part, and main couch (3%/3 mm, recorded as G0, G1, G2, and G3, in turn) sites. The decrease in the passing rate at 120° and 240° reached the maximum at the joint part, followed by the main couch, and reached the minimum at the extension plate. The four angles had the lowest pass rates at the joints (P<0.01). At the commonly used 3% 3 mm gamma pass rate condition, there was no statistical difference in the extension plate value compared to the reference value; similarly, there was no statistical difference in the main couch value compared to the reference value.

**Table 2:** Passing rates of the NPC IMRT plan beams after passing through the extension plate, joint, and main couch plate.

Gantry (°)	G1 (mean±SD)	G2 (mean±SD)	G3 (mean±SD)	G0 (mean±SD)	P1	P2	P3
160	99.68±0.16	97.34±1.62	99.32±0.34	99.51±0.45	0.23	≤0.01	0.30
120	98.87±1.12	84.91±3.62	96.80±3.34	99.60±0.17	0.07	≤0.01	0.03
240	99.16±0.75	86.77±2.79	97.50±2.23	99.29±0.55	0.03	≤0.01	0.12
200	99.56±0.17	96.79±0.92	99.12±0.48	99.28±0.38	0.57	≤0.01	0.02

P1, P2, and P3 are P-values for each group compared with the reference values.

## Discussion

The carbon fiber treatment couch is an indispensable fixing device during radiotherapy that also affects the radiation field dose [7]. The carbon fiber treatment couch, if properly positioned, has no embedded metal in the treatment area and has good radiation transmission. The maximum attenuation coefficient of 6 MV X-rays passing through the treatment couch vertically is 2.4%, which meets the clinical requirements of IMRT [15]; however, not all radiation fields are able to meet this requirement. For radiation fields with different gantry angles, the attenuation is approximately 3%–6%. To reduce the dose attenuation error caused by the treatment couch, the dose attenuation of the treatment couch needs to be corrected [16, 17]. Currently, most treatment plan designers use actual measurement data or add a virtual treatment couch model to the treatment plan to correct the effect of dose attenuation. Due to positioning error infractions and lack of information about the actual couch position in the planning CT images, even if the TPS virtual treatment couch model is added, the dose effect of the treatment couch on the irradiation field cannot be truly determined.

This study provides actual measurement, TPS plans calculation, and dose verification to study the effect of the treatment couch on the radiation field dose. Both the measured results and the TPS calculations included the attenuation effects and scattering contributions from the treatment couch. Because the measurement of the radiation dose was performed on a real treatment couch, the treatment couch scattered radiation was already acting on the detector. The planned system modeling process included the scattering effect part. Thus, the treatment couch dose calculation process already included the effect of scattered radiation. Therefore, the effect of scattering on dose has not been discussed separately. The effect on the dose was a result of combining attenuation and scattering. The Monaco version (5.11.03) contains only the main couch base model of the virtual treatment couch, without the other treatment couch parts since only the main couch base can be added when creating a virtual treatment couch. In future versions, Elekta may develop a model containing the joint and extensions of the treatment couch. Furthermore, it is necessary to point out that the attenuation and scattering of the dose by the treatment couch cannot be ignored and thus should be considered in the design of the treatment plan. It is necessary to add an accurate treatment couch model and ensure the relative position of the patient and the couch.

It could be seen from the measurement results and statistical analysis in Table 1 that the treatment couch has obvious dose reduction. It should also be pointed out that since the measured value was the value of the effective point, it was impossible to determine the effective point in the planning system. If the effective point was located at a point, the calculated value fluctuates greatly, leading to inaccurate results. Instead, we chose to outline all sensitive volumes of the air ionization chamber and used its mean value to represent the effective point dose. Therefore, the calculated value cannot be directly compared with the measured value. We guarantee that intra-group comparisons can be made by measurement or calculation under the same conditions.

As shown in Fig 5, the measured values were most consistent with the attenuation of the values calculated from the CBCT images as they almost overlapped. Within the range of -90° to 90° of the gantry angle, the dose attenuation was almost 0 because the beams did not pass through and were not affected by the couch. With the increase in the distance between the beam and treatment couch, the dose attenuation curves of the measured values and the actual couch calculated by the TPS were significantly increased near the angles of 110° and 250°, indicating that the dose attenuation at the joint was much larger than that at other positions. However, the dose distribution curve near the two angles of 110° and 250° is convex, and the dose attenuation at the joint is much larger than that in other locations. The effect of the treatment couch on the dose is highly correlated with the angle of the gantry, and the attenuation of the dose increases sharply at approximately 110° and 250° [18]. In the actual plan design and treatment process, most radiotherapy physicists and technologists do not consider the effect of the couch joint on the dose attenuation [19]. Fig 6A–6D present the measured and calculated values of the detector at different positions normalized to the reference position. The measured values at the extension plate, joint part, main couch, and actual couch dose calculated by the TPS were almost symmetrically distributed, with the dose distribution curve of both near the two positions of 110° and 250° being concave. During the gradual rotation of the gantry from 100° to 260°, the dose attenuation increased first, reached the maximum value, and then gradually decreased, reaching the minimum value at 180°, and then increased and decreased symmetrically, with the dose attenuation being the largest at the junction. Fig 6B–6D show the comparison between the TPS with added virtual treatment couch model and the actual couch dose attenuation calculated by the TPS. They show that the TPS virtual treatment couch model cannot fully simulate the attenuation of the actual treatment couch, although the maximum attenuation angles are similar both at 110° and 250°. The maximum dose attenuation factor of the virtual couch model calculated by the TPS was 4.0%, while the maximum dose attenuation factor of the actual couch calculated by the TPS was 33%, which was much larger than the attenuation of the TPS virtual couch model. As shown in Fig 5, the measured value and actual couch dose attenuation calculated by the TPS were much larger than those in other cases and were also quite close, indicating that the actual couch dose attenuation calculated by the TPS could truly reflect the true attenuation of the actual couch. As shown in Fig 1, the Elekta treatment couch is composed of an extension plate, a joint, and the main couch. In particular, the joint between the main couch and the extension plate is connected by a hook and has a reinforcement structure. This high-density reinforced structure can be observed on a CBCT image (Fig 2). The thickness of the extension plate is thinner than that of the treatment couch, and the attenuation of X-rays is smaller than that of the main treatment couch, conforming to the law of X-ray attenuation. The ray must pass through the hook and the reinforcement material at the two angles of 110° and 250° at the junction first. The dose attenuation coefficient at this place is significantly higher than that at other positions, which causes the measured value of the ionization chamber to be low. Therefore, it is necessary to avoid rays passing through both sides of the joint, specifically at the irradiation field near 110° and 250° of the joint. Thus, when facing an unavoidable long target area, it is necessary to avoid irradiating the field through that angle. As shown in Table 2, the attenuation of the passing rate by the treatment couch was consistent with the trend of the absolute dose. The attenuation of the dose at the joint near the gantry angles of 110° and 250° is higher than that at other angles, with a deviation of 12%–15%, which is not suitable for treatment. The impact on the dose in the remaining angle intervals is close to that of the main treatment couch, and these can still be used as treatment areas. The structure and thickness of the long extension couch are not very different from the main couch; therefore, we chose the short extension plate for comparison. In the actual positional operation, we can choose the treatment couch position for different radiotherapy sites, but the dose attenuation of the treatment couch for a specific angle is relatively large, and the attenuation from that angle is not negligible, even with the VMAT technique.

In summary, during the planning and execution of radiotherapy treatment, the positional relationship between the target area and the treatment couch should be considered comprehensively. The appropriate treatment angle should be selected, and attention should be paid to avoiding areas with large dose attenuation. Reducing the impact of the treatment couch on the planned dose is essential to ensuring overall dose accuracy.

## Supporting information

S1 Data (XLS)
